# Comparative analysis of stomatal pore instance segmentation: Mask R-CNN vs. YOLOv8 on Phenomics Stomatal dataset

**DOI:** 10.3389/fpls.2024.1414849

**Published:** 2024-12-06

**Authors:** Thanh Tuan Thai, Ki-Bon Ku, Anh Tuan Le, San Su Min Oh, Ngo Hoang Phan, In-Jung Kim, Yong Suk Chung

**Affiliations:** ^1^ Department of Plant Resources and Environment, Jeju National University, Jeju, Republic of Korea; ^2^ Multimedia Communications Laboratory, University of Information Technology, Ho Chi Minh City, Vietnam; ^3^ Multimedia Communications Laboratory, Vietnam National University, Ho Chi Minh City, Vietnam; ^4^ Department of Electrical and Computer Engineering, Iowa State University, Ames, IA, United States; ^5^ Faculty of Biology and Biotechnology, University of Science, Ho Chi Minh City, Vietnam; ^6^ Department of Horticulture, Jeju National University, Jeju, Republic of Korea; ^7^ Faculty of Biotechnology, Bio-Resources Computing Research Center, Jeju National University, Jeju, Republic of Korea; ^8^ Phytomix Corporation, Jeju, Republic of Korea

**Keywords:** stomata, phenotyping, instance segmentation, Mask-RCNN, YOLO

## Abstract

This study conducts a rigorous comparative analysis between two cutting-edge instance segmentation methods, Mask R-CNN and YOLOv8, focusing on stomata pore analysis. A novel dataset specifically tailored for stomata pore instance segmentation, named PhenomicsStomata, was introduced. This dataset posed challenges such as low resolution and image imperfections, prompting the application of advanced preprocessing techniques, including image enhancement using the Lucy-Richardson Algorithm. The models underwent comprehensive evaluation, considering accuracy, precision, and recall as key parameters. Notably, YOLOv8 demonstrated superior performance over Mask R-CNN, particularly in accurately calculating stomata pore dimensions. Beyond this comparative study, the implications of our findings extend across diverse biological research, providing a robust foundation for advancing our understanding of plant physiology. Furthermore, the preprocessing enhancements offer valuable insights for refining image analysis techniques, showcasing the potential for broader applications in scientific domains. This research marks a significant stride in unraveling the complexities of plant structures, offering both theoretical insights and practical applications in scientific research.

## 1 Introduction

Stomata, microscopic pores found on the surfaces of plant leaves, stems, and other plant organs, play a pivotal role in plant physiology (Fetter et al., 2019). These small openings facilitate gas exchange, enabling plants to take in carbon dioxide essential for photosynthesis and release oxygen and water vapor (Evans and Von Caemmerer, 1996). Studying stomatal behavior and density is crucial in understanding plant responses to environmental factors such as light intensity, humidity, and carbon dioxide levels. Additionally, stomatal density is a key indicator of a plant’s adaptation to various ecological niches and climate conditions (Hetherington and Woodward, 2003).

Traditionally, stomata analysis has been a labor-intensive and time-consuming task, often performed manually by plant biologists (Jayakody et al., 2017). The advent of computer vision and deep learning techniques has revolutionized this process, offering automated and efficient solutions for stomata segmentation in plant images. The development of robust and accurate algorithms for stomata segmentation not only expedites research in plant physiology but also holds significant implications for agriculture, environmental science, and climate change studies (Hetherington and Woodward, 2003).

Semantic segmentation, a critical task in computer vision, has seen significant advancements with the development of deep learning techniques (Lateef and Ruichek, 2019). Early methods relied on hand-crafted features, but the introduction of convolutional neural networks (CNNs) revolutionized the field (Li et al., 2022). Fully Convolutional Networks (FCNs) (Long et al., 2015) were among the first deep learning models to achieve pixel-level classification by replacing fully connected layers with convolutional layers, enabling end-to-end learning for segmentation tasks. Building upon this, U-Net (Ronneberger et al., 2015) introduced a more robust encoder-decoder architecture, enhancing segmentation performance in biomedical imaging by combining low-level spatial information with high-level feature representations. Meanwhile, Mask R-CNN (He et al., 2017) and YOLOv8 (Jocher, 2023) have emerged as powerful instance segmentation algorithms, demonstrating outstanding performance in various object detection tasks (Jocher, 2023). However, their application and effectiveness in stomata segmentation re-main relatively unexplored. This study aims to bridge this gap by conducting a comprehensive comparative analysis of these algorithms in the context of stomata segmentation. By evaluating their accuracy, speed, and adaptability to the complex biological structures of stomata, this research contributes valuable insights to the field of plant biology and automated phenotyping. Importance of stomata segmentation in plant biology.

**Figure 1:**
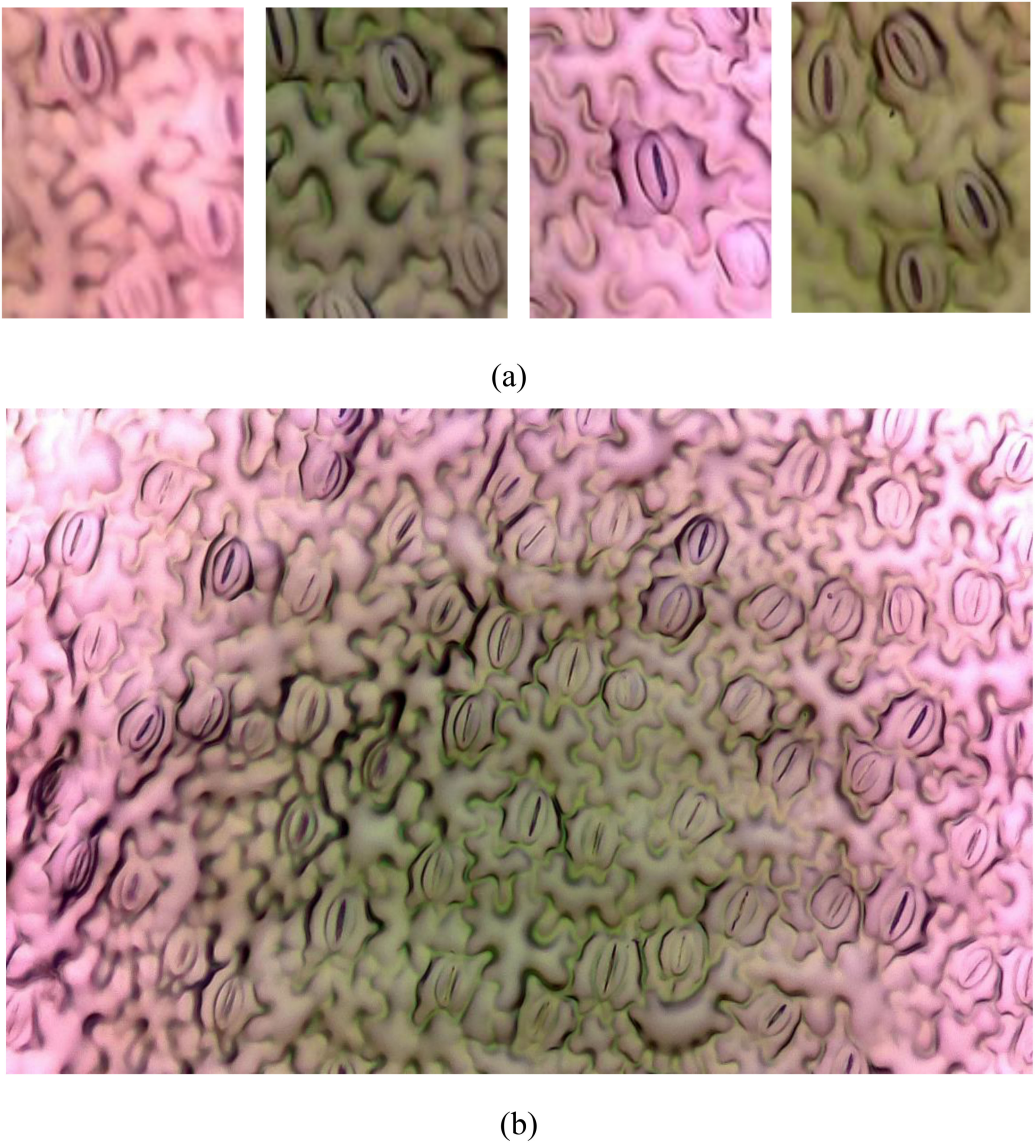
Image from the PhenomicsStomata dataset. **(A)** Splitted images, **(B)** Original full-sized image.

In the field of plant biology, the precise quantification and analysis of stomatal pores are of fundamental importance for understanding plant physiology and responses to environmental stimuli (Jayakody et al., 2021). Stomata, serving as the primary conduits for gas exchange in plants, play a pivotal role in essential processes such as photosynthesis and transpiration. Traditional methods of stomatal analysis are laborious and time-intensive, underscoring the necessity for advanced automated techniques to streamline the research process (Duarte et al., 2017). The challenge at hand involves developing an automated system capable of accurately identifying and segmenting individual stomata from intricate plant imagery. This task is intricate due to the inherent variability in stomatal shapes, sizes, and orientations across different plant species and environmental conditions. Additionally, achieving high seg-mentation accuracy is paramount for ensuring reliable quantitative analysis, significantly impacting fields such as plant physiology, agriculture, and climate change research (Bheemanahalli et al., 2021). **Figure 1** illustrates examples of stomatal images captured under a microscope. **Figure 1B** displays a full-sized image, while **Figure 1A** showcases four split images, highlighting the complexities involved in the segmentation process.

This study addresses the challenge by employing cutting-edge deep learning algorithms, Mask R-CNN and YOLOv8, for automated stomata segmentation on the novel dataset PhenomicsStomata. Through rigorous experimentation and comparative analysis, the study aims to assess the efficacy of these algorithms in accurately delineating stomatal structures from high-resolution plant images. Specific objectives include optimizing algorithmic parameters for enhanced accuracy, evaluating the algorithms’ robustness across diverse plant species, and elucidating the algorithms’ computational efficiency for real-time applications. By achieving these objectives, this research provides a robust, automated solution for stomata segmentation, thereby advancing the frontiers of plant biology re-search, particularly on the PhenomicsStomata dataset. Furthermore, the insights gained from this study can catalyze advancements in automated phenotyping, contribute to a deeper understanding of plant-environment interactions, and inform sustainable agricultural practices and environmental conservation efforts.

In contemporary computer vision, Mask R-CNN (Mask Region-based Convolutional Neural Network) and YOLOv8 (You Only Look Once version 8) stand as prominent pillars of object detection and instance segmentation methodologies. Mask R-CNN, an extension of Faster R-CNN (Ren et al., 2015), excels in accurately delineating object boundaries while simultaneously providing per-pixel segmentation masks. This unique capability makes it particularly potent for tasks requiring precise localization and intricate segmentation, such as stomata analysis in plant biology. Mask R-CNN achieves this through a two-stage process: first, a Region Proposal Network (RPN) generates potential bounding box proposals, and subsequently, these proposals undergo refinement through convolutional layers for both bounding box regression and mask prediction. The architecture’s effectiveness lies in its ability to seamlessly fuse object localization and segmentation, offering a holistic solution for tasks demanding detailed object understanding. On the other front, YOLOv8 epitomizes the single-shot object detection paradigm. Its hallmark feature is real-time processing, providing rapid predictions while maintaining robust accuracy. YOLOv8 employs a unified neural network to predict bounding boxes and class probabilities directly from the entire image. The architecture’s efficiency is attributed to its division of the input image into a grid, with each grid cell responsible for predicting objects. This grid-based approach, coupled with feature pyramid networks, enables YOLOv8 to swiftly process diverse object scales and sizes. Both Mask R-CNN and YOLOv8 have played pivotal roles in revolutionizing object detection, impacting fields ranging from autonomous vehicles to biomedical imaging. In the domain of stomata segmentation, their prowess is harnessed to automate the meticulous task of isolating these vital plant structures, fostering advancements in plant biology and phenotypic analysis. The amalgamation of advanced convolutional architectures, strategic neural network designs, and innovative training techniques renders Mask R-CNN and YOLOv8 indispensable tools in the contemporary landscape of computer vision applications. It’s crucial to underline that this study did not undertake modifications to the fundamental structures of Mask R-CNN or YOLOv8. The research approach centered on the utilization of the official architectures of these models tailored specifically for the PhenomicsStomata dataset. The study refrained from altering the core frameworks of Mask R-CNN or YOLOv8, emphasizing the use of their original designs. For a comprehensive understanding of these deep learning models and their intricate components, readers are encouraged to refer to the related literature, where detailed insights into these models and their inherent mechanisms are provided. This strategic decision ensured the study’s focus remained on the adaptation and application of these established models to the unique challenges posed by the PhenomicsStomata dataset.

## 2 Materials and methods

### 2.1 Image acquisition and preprocessing steps

In the pursuit of precise stomata segmentation, the acquisition and preprocessing of plant images form the foundational bedrock of our study. The images, sourced from leaves of Hedyotis corymbosa, are cultivated under controlled conditions to ensure uniformity and eliminate extraneous variables. Germinated in a substrate blend of clean soil and cow manure, the plants are meticulously nurtured in a greenhouse under specific light conditions—450 ± 100 μmol.m−2.s−1 sunlight at 32 ± 2°C and 70 ± 5% relative humidity.

Following a standardized protocol, the fifth leaves from the top of the plants are delicately affixed onto microscope slides using cyanoacrylate glue. The resulting leaf surfaces are meticulously captured using a CKX41 inverted microscope equipped with a DFC450 camera, generating high-resolution images stored in JPEG format at 2592 × 1458 pixels.

**Figure 2:**
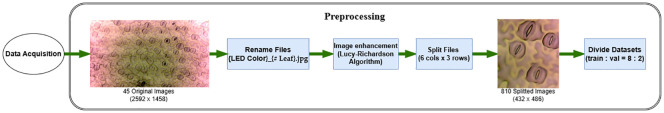
Preprocessing pipeline of the dataset.

Addressing the challenge of blurriness in stomatal images, we employ the Lucy-Richardson Algorithm (Fish et al., 1995), known for its deblurring capabilities as show in **Figure 2**. Applied iteratively to each image, this algorithm refines the images, reducing blurriness and enhancing the definition of stomatal boundaries. The application of this algorithm is grounded in scientific principles, ensuring a rigorous enhancement process.

The original image filenames are systematically replaced with a standardized convention {W}_{Leaf Number}.jpg, with ‘W’ signifying ‘White.’ To optimize subsequent detection processes, the enhanced images undergo strategic segmentation into smaller, non-overlapping segments. Using a grid-based approach, the images are divided into 6 columns and 3 rows. This meticulous segmentation results in 18 focused images, each containing a subset of stomatal pores. This precision-driven approach ensures well-defined regions of interest, facilitating precise and efficient detection in subsequent stages.

To mitigate computational complexities and streamline subsequent analysis, these split images are resized to 512×512 pixels, reducing memory overhead while preserving pertinent morphological information. This resizing, from the original 432×486 pixels, proves instrumental in enhancing the efficiency of subsequent deep learning algorithms.

The significance of this preprocessing methodology lies not only in its role as a computational optimization strategy but also in its meticulous preservation of stomatal morphology. The resultant dataset, meticulously preprocessed to encapsulate the nuances of stomatal structures, forms the nucleus upon which our subsequent segmentation methodologies are anchored, ensuring an empirical and precise approach in our pursuit of automated stomata analysis.

The prepared dataset is partitioned into distinct subsets for training and validation, maintaining a balanced ratio of 8:2. This division exposes machine learning models to diverse yet representative data. The validation set, constituting 20% of the data, serves as an independent benchmark, preventing overfitting and ensuring the robustness of the trained models.

### 2.2 Instance segmentation using Mask R-CNN and YOLOv8

Both Mask R-CNN and YOLOv8 were trained using the official configurations recommended by the developers of the models, without any modifications to their architectures. For Mask R-CNN, a learning rate of 0.001 was used with a batch size of 8, and the model was trained for 50 epochs (**Figure 7**). For YOLOv8, the learning rate was 0.01, with a batch size of 16, and the model was also trained for 100 epochs (**Figure 8**).

**Figure 3:**
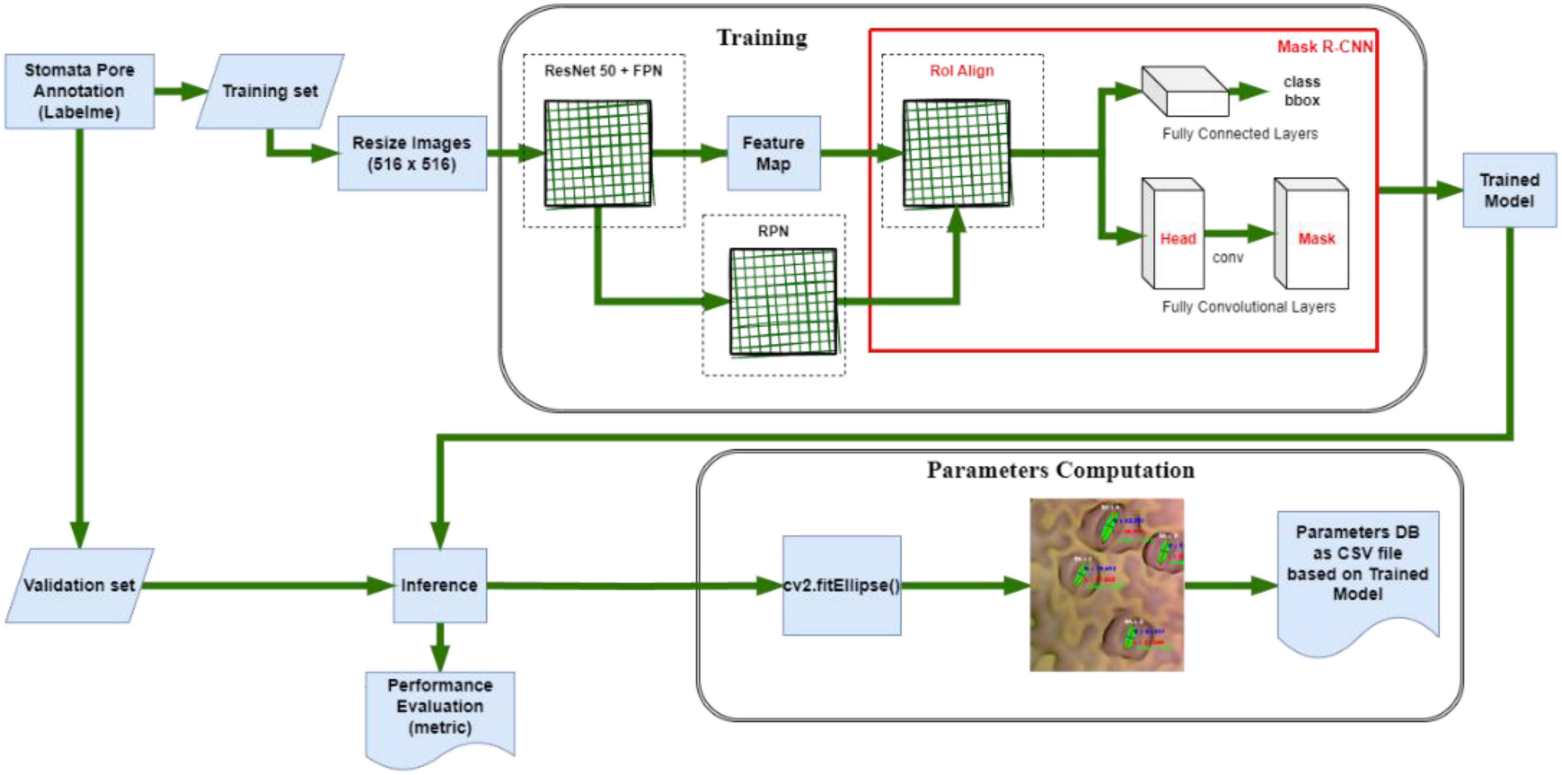
Pipeline of Mask R-CNN for instance segmentation on Stomata pore dataset.

The training phase of Mask R-CNN, a cutting-edge instance segmentation algorithm, demands a meticulous amalgamation of neural network architecture and hyperparameter tuning (**Figure 3**). In this study, Google Colab (“Google Colab, Https://Colab.Google/” 2023), bolstered by an NVIDIA Tesla T4 GPU, serves as the computational powerhouse, accelerating the intricate computations involved in training. This configuration ensures that the model is adeptly harnessed to process the complexities of stomatal structures in plant imagery. Crucial to the training process is the compatibility of software components. To safeguard against discrepancies during inference, we meticulously downgraded TensorFlow (Abadi et al., 2016) from version 2.9.2 (released on January 4, 2023) to version 2.5.0. Accompanying this was a corresponding downgrade of the cuDNN library to version 8.1.0.77-1+cuda11.2. This meticulous version control guarantees a seamless interplay between the software components, mitigating potential errors and ensuring the model’s precision during subsequent inference. The implementation of Mask R-CNN in this study originates from the Matterport library (Abdulla, 2023), renowned for its efficacy in complex object detection tasks. However, adapting this architecture to stomata detection necessitates fine-tuning. To tailor the model to our specific task, we initiated the training process with a dataset comprising 810 meticulously annotated images. This dataset was strategically divided into train and validation sets, allocating 648 images for training and 162 for validation. This partitioning ensures a robust training regimen while enabling a stringent evaluation of the model’s performance.

In defining the confidence threshold for object detection, a critical parameter in the process, a judicious choice was made to set it at 0.5. This threshold serves as the discriminatory metric, determining the minimum confidence score required for an object to be classified as detected (He et al., 2017). A balanced selection, this threshold strikes an equilibrium between precision and recall, essential in optimizing the algorithm’s accuracy without incurring undue compromises in detection sensitivity. Through this meticulous orchestration of computational resources, software compatibility, and parameter tuning, the training process of Mask R-CNN converges towards a model finely attuned to the intricate task of stomata segmentation. This calibrated model forms the linchpin of our automated stomata analysis, promising not just accuracy, but also reliability of plant biology research.

YOLOv8 (Jocher, 2023) stands as a paradigm of efficiency, offering real-time predictions while maintaining commendable accuracy. The training process for YOLOv8 demands meticulous attention to architectural nuances and strategic parameter configurations to harness its potential effectively. In this study, the training endeavors commence on a robust computational platform, leveraging a system fortified with a Windows 11 Professional 64-bit operating system, an AMD Ryzen 7 5800x3D 8-Core Processor, and 32GB of RAM. Additionally, the training benefits from the computational prowess of an NVIDIA GeForce RTX 4080 GPU with 16GB of dedicated memory. This hardware synergy forms the cornerstone for expedited computations, enabling the model to grapple with the intricacies of stomatal detection in a diverse array of plant imagery.

**Figure 4:**
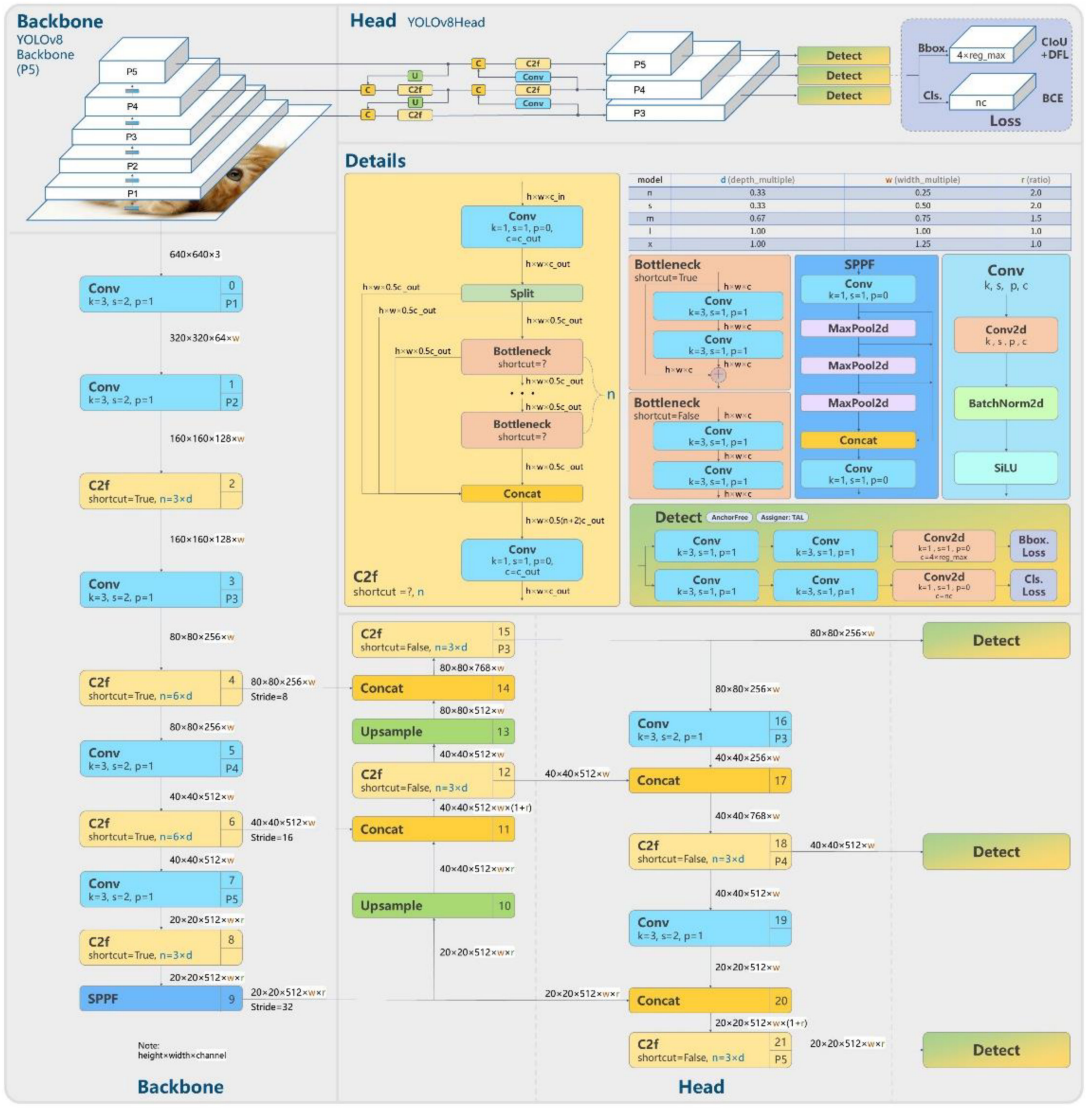
YOLOv8 structure (“Model Structure of YOLOv8, Https://Github.Com/RangeKing”, 2024).

In this study, we have strategically utilized the meticulously crafted official version of YOLOv8, a sophisticated deep learning architecture as show in **Figure 4**, to tackle the intricate task of stomatal segmentation. YOLOv8’s exceptional efficiency in processing images in a single pass is a testament to its computational prowess. The underlying neural network architecture of YOLOv8 is ingeniously engineered, incorporating advanced features such as feature pyramid networks and grid-based object detection techniques. Through a meticulous parameter configuration process, where learning rates and batch sizes are finely tuned, the model strikes an optimal balance between rapid convergence and stable performance. The iterative training process, crucial for enhancing predictive capabilities, involves exposing the model to a meticulously curated dataset rich in stomatal annotations. This dataset diversity enables YOLOv8 to discern the subtle nuances within various stomatal instances, ensuring its adaptability to the complexities of natural plant imagery. This calibrated approach represents a seamless fusion of hardware resources, architectural intricacies, and parameter optimization, resulting in a YOLOv8 model uniquely poised to decipher the complexities of stomatal segmentation. Its real-time predictive abilities and remarkable accuracy position it as a powerful tool in the domain of automated plant phenotyping, promising significant contributions to the fields of plant biology and automated image analysis.

**Figure 5:**
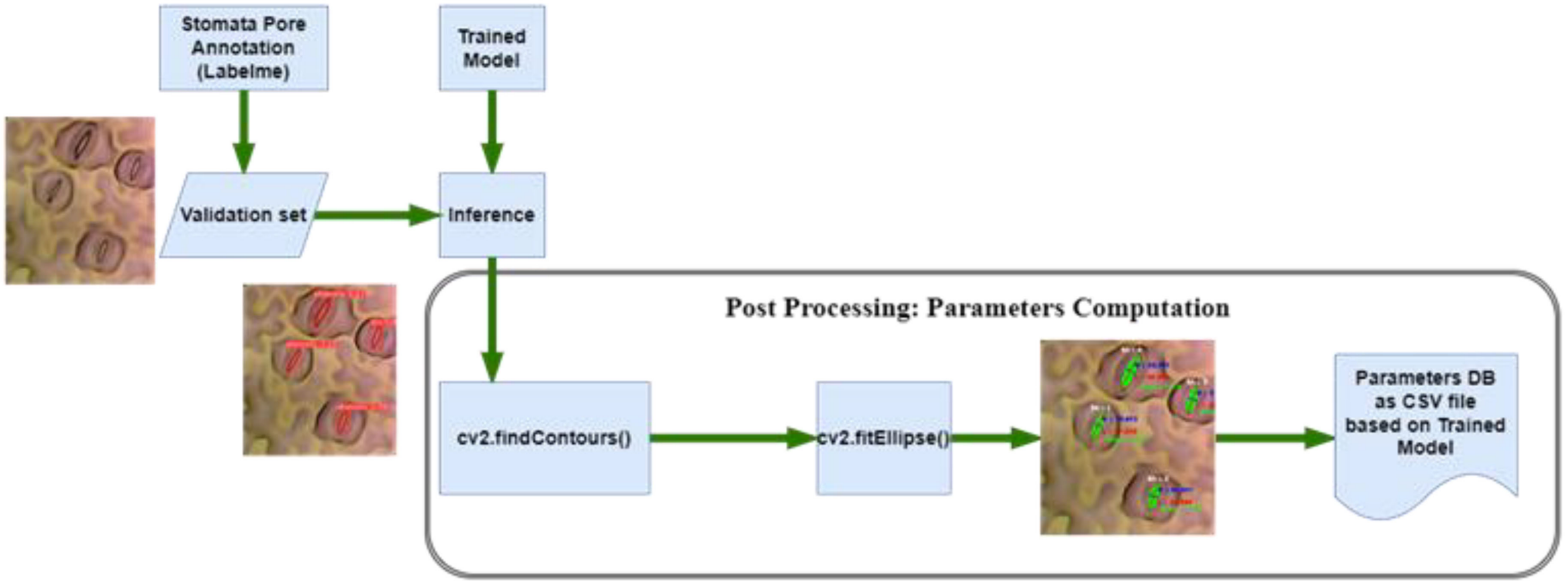
Post processing for parameter computation.

In the domain of image analysis and segmentation, the extraction of mask contours constitutes a pivotal step, especially when dealing with intricate biological structures like stomata. Following the instance segmentation process, wherein Mask R-CNN or YOLOv8 generates precise masks delineating stomatal regions, the subsequent task involves extracting these masks’ contours. Utilizing the sophisticated capabilities of the OpenCV (“OpenCV, Https://Opencv.Org/”, 2023) library, specifically the findContours function, the algorithm meticulously traces the perimeters of the generated masks. These contours represent the precise boundaries of stomatal pores, capturing their intricate shapes with sub-pixel accuracy. The findContours function operates on binary images, identifying connected components and outlining their boundaries. In the context of stomatal segmentation, the binary masks generated by the algorithms serve as the input, containing pixel values denoting stomatal and non-stomatal regions as shown in **Figure 5**. The function systematically traces along the pixels’ edges, discerning the transitions from stomatal to non-stomatal areas. As a result, a series of coordinates representing the contour’s path are derived, encapsulating the stomatal region’s geometry. This contour extraction process is not merely a technicality; it represents the bridge between raw pixel data and meaningful geometric information. These contours, essentially a sequence of points, serve as the foundation for subsequent analyses, facilitating computations of stomatal parameters such as area, width, length, and angle.

The precision of this contour extraction process significantly impacts the accuracy of subsequent analyses. Imperfections or inaccuracies in contour delineation can lead to distorted measurements, thereby affecting the reliability of the derived stomatal parameters. Hence, the careful implementation of the findContours function, coupled with the high-quality masks generated by the segmentation algorithms, ensures the faithful representation of stomatal contours. This meticulous process forms the cornerstone for quantitative analyses, enabling precise morphological characterizations crucial for advancing our understanding of plant physiology and environmental responses. Following the meticulous extraction of mask contours, the subsequent step involves the computation of essential stomatal parameters, providing quantitative insights into these biological structures. Leveraging the rich functionality of the OpenCV library, particularly the cv2.fitEllipse function, and employing mathematical formulations, our analysis delves into the intricate morphological characteristics of stomata. The cv2.fitEllipse function operates on the extracted contours, fitting an ellipse that best approximates the stomatal shape. This elliptical approximation yields crucial parameters: the center coordinates, major and minor axis lengths, and the orientation angle. These parameters encapsulate the inherent geometric properties of stomata, enabling a comprehensive characterization beyond mere visual segmentation.

From these fundamental parameters, additional significant measures are derived. The width and length of stomatal pores are calculated, providing insights into their structural dimensions. The area of each stomatal pore, a pivotal metric reflecting its size, is computed as well. Additionally, employing trigonometric principles, the diameter of the stomatal pore is accurately determined. The application of sine and cosine functions, utilizing the center coordinates and the minor axis length, yields the diameter, a fundamental parameter crucial for various physiological analyses. These computations are not confined to individual stomata; rather, they extend across the entire dataset, generating a wealth of quantitative data. The precise values acquired through these computations serve as quantitative descriptors, enabling comparative analyses between stomatal populations across diverse plant species or experimental conditions. The accuracy and precision of these parameters are contingent on the fidelity of the extracted contours. Thus, the robustness of the cv2.fitEllipse function in capturing the true stomatal geometry, combined with the high-quality masks generated during segmentation, ensures the reliability of the computed parameters. These meticulously computed stomatal parameters provide a quantitative foundation for subsequent statistical analyses, fostering a deeper understanding of stomatal behavior and adaptation in the plant biology.

### 2.3 Model evaluation and performance metrics

**Figure 6:**
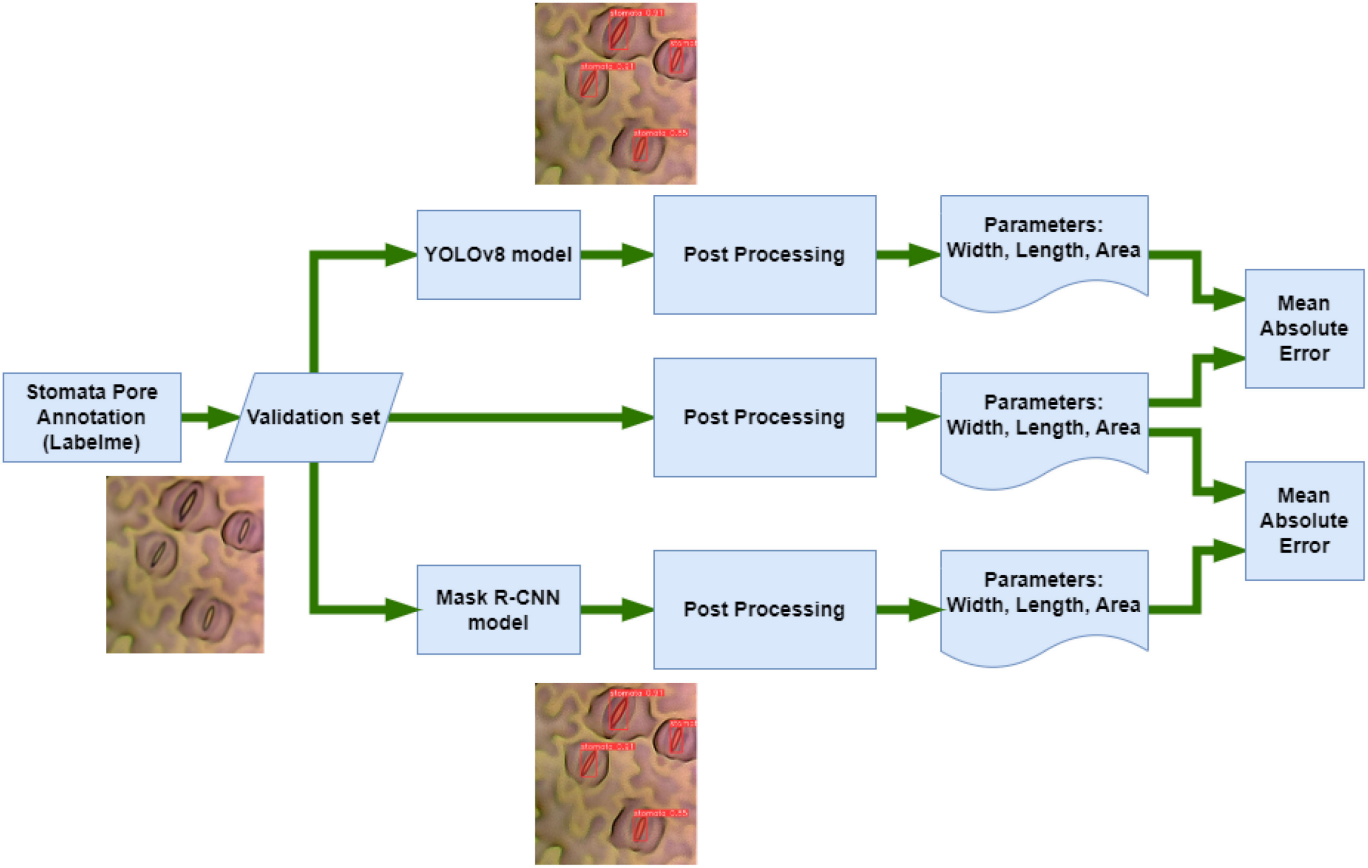
Model evaluation diagram.

A meticulous evaluation of model performance against ground truth annotations is paramount for validating the accuracy and reliability of segmentation algorithms as shown in **Figure 6**. The ground truth, representing manually annotated stomatal pores, serves as the reference against which the predictions of Mask R-CNN and YOLOv8 are assessed. Ground truth annotations, meticulously created through manual delineation, epitomize the precise location and boundaries of stomatal pores. These annotations encapsulate the gold standard against which automated segmentation methods are benchmarked. The ground truth provides a clear, human-verified delineation of each stomatal pore, offering a reliable foundation for comparative analysis. Mask R-CNN, a sophisticated instance segmentation algorithm, generates predictions by outlining stomatal pores based on learned patterns from the training data. Its outputs consist of bounding boxes and pixel-wise masks encapsulating the segmented stomatal regions. The algorithm aims to align its predictions with the ground truth, with a focus on accurate boundary delineation and shape consistency. YOLOv8, a state-of-the-art object detection framework, predicts stomatal pores using bounding boxes coupled with confidence scores and semantic segmentation within each detected region. Known for its efficiency in real-time detection tasks, YOLOv8 delivers fast and accurate predictions. Similar to Mask R-CNN, its outputs are assessed against ground truth annotations, with key performance metrics including precision, recall, and intersection over union (IoU), allowing a comprehensive evaluation of the model’s segmentation accuracy. Comparing the outputs of Mask R-CNN and YOLOv8 against the ground truth enables a comprehensive assessment of their segmentation accuracy. Metrics such as intersection over union, precision, and recall are computed to quantitatively measure the alignment between the predicted stomatal pores and the ground truth annotations. The comparative analysis sheds light on the strengths and limitations of each algorithm, offering insights into their respective capabilities in accurately capturing the intricate features of stomatal pores. This rigorous comparison not only validates the effectiveness of the segmentation algorithms but also aids in identifying areas of improvement, guiding further refinements in these methodologies for enhanced precision and reliability in the intricate task of stomatal pore segmentation.

**Figure 7:**
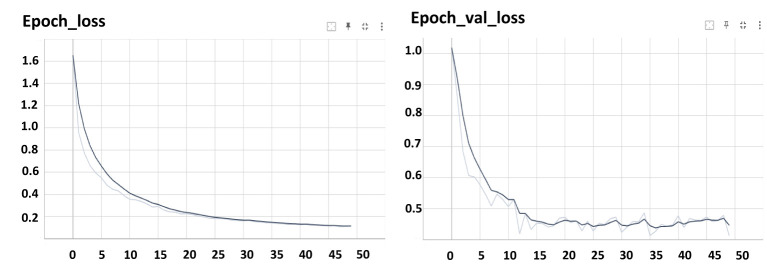
Loss of the training Mask R-CNN.

**Figure 8:**
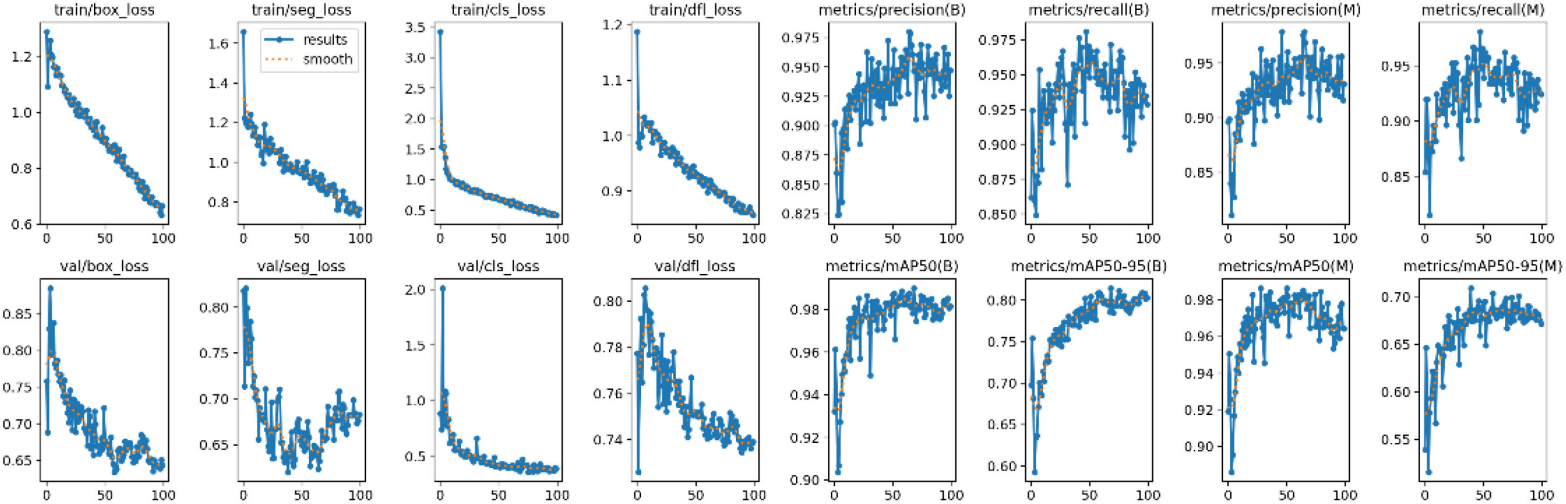
Training log of YOLOv8 on dataset.

A suite of evaluation metrics is instrumental in quantifying the accuracy and efficacy of segmentation algorithms. These metrics, including Intersection over Union (IoU) (He et al., 2017), Precision, Recall, and F1 Score (Saito and Rehmsmeier, 2015), provide a nuanced understanding of how well the algorithmic predictions align with the ground truth annotations.


(1) $$IOU=\frac{Area\left(GTBbox\cap PBbox\right)}{Area\left(GTBbox\cup PBbox\right)}$$

(2) $$Precision=\frac{True\;Positive}{True\;Positive\;+\;False\;Positive}$$

(3) $$Recall=\frac{True\;Positive}{True\;Positive\;+\;False\;Negative}$$

(4) $$F1=2*\;\frac{Precision*Recall}{Precision+Recall}$$

IoU, also known as the Jaccard Index, measures the overlap between the predicted segmentation mask and the ground truth mask. It is computed as the ratio of the intersection area to the union area of the predicted and ground truth regions. A higher IoU signifies a better match between the prediction and the actual segmentation, indicating a precise delineation of object boundaries. Precision quantifies the accuracy of positive predictions made by the model. It is calculated as the ratio of true positive predictions to the total positive predictions (true positives and false positives). Precision is vital in tasks where false positives need to be minimized, ensuring that the predicted positive instances are re-liable and accurate. Recall, also known as sensitivity or true positive rate, assesses the model’s ability to capture all positive instances. It is calculated as the ratio of true positive predictions to the total actual positives (true positives and false negatives). Recall is crucial when missing positive instances is highly undesirable, emphasizing the model’s sensitivity to detect all relevant objects. The F1 Score strikes a balance between precision and recall, offering a single metric that encapsulates both aspects. It is the harmonic mean of precision and recall and provides a comprehensive evaluation of the model’s performance. A higher F1 score indicates a well-rounded model that achieves both high precision and recall, highlighting its effectiveness in accurate object segmentation. These metrics collectively provide a thorough assessment of the segmentation algorithm’s performance. IoU ensures accurate boundary delineation, precision emphasizes precise positive predictions, recall focuses on exhaustive detection of positive instances, and F1 score balances the trade-off between precision and recall. By meticulously computing and interpreting these metrics, researchers gain valuable insights into the algorithm’s strengths and areas for improvement, fostering the continual advancement of image segmentation methodologies.

Visualizations play a pivotal role in comprehending the intricate dynamics of model predictions. These visual representations, coupled with qualitative analysis, offer invaluable insights into the nuances of segmentation algorithms, shedding light on both their successes and challenges. One fundamental aspect of qualitative analysis involves visualizing the masks generated by the segmentation models. These masks, outlining the segmented regions, are overlaid onto the original images. By juxtaposing the predicted masks with the input images, researchers gain a firsthand understanding of the model’s ability to capture the subtle details of stomatal pores. Visual inspection aids in identifying discrepancies between the predicted boundaries and the actual stomatal structures, facilitating a nuanced assessment of segmentation accuracy. Comparing the visual outputs of different segmentation algorithms, such as Mask R-CNN and YOLOv8, alongside the ground truth annotations, forms the cornerstone of qualitative analysis. Researchers meticulously scrutinize these visual comparisons to discern similarities and disparities. Examining in-stances where algorithms excel or falter provides qualitative context to quantitative metrics. Such comparative visual analyses are instrumental in pinpointing specific scenarios, such as challenging lighting conditions or overlapping stomatal structures, where algorithms might exhibit varying degrees of accuracy. Visualizations aid in identifying false positives (erroneously predicted stomatal pores) and false negatives (missed stomatal pores) within the model outputs. These mispredictions are scrutinized to discern patterns and commonalities, offering cues for potential model enhancements. By closely analyzing these misclassifications, researchers can iteratively refine algorithms, addressing specific challenges encountered during segmentation tasks.

Beyond mere pixel-level accuracy, visualizations provide an intuitive understanding of the clarity and interpretability of segmented stomatal pores. Researchers assess the smoothness of segmented boundaries, the level of detail captured, and the overall visual fidelity of predictions. These qualitative aspects, often nuanced and challenging to quantify, are pivotal in gauging the real-world applicability of segmentation models, especially in scientific contexts where precise stomatal measurements are crucial. The visualizations and qualitative analyses not only serve as tools for model refinement but also offer scientific insights into plant biology. Researchers can leverage these visual outputs to study stomatal behavior under varying environmental conditions. Observations regarding stomatal opening and closure, density, and arrangement within leaves can be derived, contributing to a deeper understanding of plant physiological responses. Visualizations, therefore, bridge the gap between technical algorithmic performance and meaningful biological interpretations, fostering a holistic approach to image analysis in botanical research.

## 3 Results

In **Figure 7**, the training and validation loss curves for Mask R-CNN illustrate the model’s learning progress. The training loss steadily decreased over epochs, reaching an impressive low of 0.16. This decrease signifies the model’s ability to effectively minimize errors during the training process. Simultaneously, the validation loss, a crucial metric indicating the model’s performance on unseen data, reached a value of 0.25. While slightly higher than the training loss, this validation loss still indicates the model’s generalization capability and its ability to perform well on new, unseen stomatal pore images. The smaller the loss value, the better the model’s accuracy and predictive power, highlighting the robustness of Mask R-CNN in accurately segmenting stomatal pores during training and validation.

**Figure 8** provides a detailed overview of the training and validation process of the YOLOv8 model on the dataset. Throughout the training phase, the model achieved remarkable progress, with the box loss reaching 0.66543, segmentation loss at 0.76429, classification loss at 0.42379, and distribution focal loss at 0.85613. These values reflect the model’s ability to optimize its predictions, ensuring precision and accuracy in identifying stomatal pores. Notably, the training phase demonstrated a high precision of 0.94709, indicating the model’s proficiency in making accurate positive predictions. The recall, signifying the model’s capability to identify all relevant instances, was at an impressive 0.9288. Additionally, the mean average precision at a confidence threshold of 0.5 reached an outstanding value of 0.98164, underlining the model’s robustness in various scenarios. Moreover, the mean average precision for confidence levels between 0.5 to 0.9 was at 0.80269, emphasizing the model’s consistent performance across different confidence ranges. During the validation phase, the model’s performance remained strong, with the box loss on the validation set measuring 0.64362, segmentation loss at 0.68271, classification loss at 0.391, and distribution focal loss at 0.7391. These validation metrics mirror the model’s ability to generalize well beyond the training data, ensuring its reliability in real-world applications. Collectively, these training and validation logs showcase the YOLOv8 model’s proficiency and reliability in accurately segmenting stomatal pores, marking a significant achievement in the botanical image analysis.

The Mean Absolute Error (Willmott and Matsuura, 2005) (MAE) serves as a critical metric, quantifying the disparity between predicted and ground truth stomatal pore attributes. Notably, **Table 1** delineates the MAE values for width, length, and area estimations, elucidating the comparative performance of Mask R-CNN and YOLOv8 against the ground truth data. In the domain of width estimation (measured in pixels), YOLOv8 exhibits a noteworthy precision, yielding an MAE of 1.83972. This marginally surpasses the MAE of 1.83979 attributed to Mask R-CNN. YOLOv8’s ability to delineate the width of stomatal pores with a slightly reduced error underscores its accuracy in capturing the fine details of these structures. When estimating the length of stomatal pores, YOLOv8 excels further, yielding an MAE of 6.19958. In stark contrast, Mask R-CNN manifests a comparatively higher MAE of 8.72383. YOLOv8’s ability to predict the length of stomatal pores with a diminished error emphasizes its proficiency in capturing the elongated dimensions of these vital biological features. The estimation of stomatal pore area, a pivotal metric in botanical research, reinforces YOLOv8’s superiority. With an MAE of 152.9066, YOLOv8 outperforms Mask R-CNN, which exhibits a higher MAE of 168.5477. YOLOv8’s adeptness in estimating stomatal pore area with reduced error signifies its precision in quantifying the surface area, crucial for various physiological analyses. These findings underscore YOLOv8’s superior accuracy in predicting width, length, and area attributes of stomatal pores when compared to Mask R-CNN. The diminished MAE values achieved by YOLOv8 highlight its robustness and efficacy in capturing the intricate morphological details of stomatal structures, thus enhancing the accuracy and reliability of botanical analyses.

**Table 1:** Mean absolute error comparison between ground truth, Mask R-CNN, and YOLOv8 outputs.

	Width(pixel)		Length(pixel)		Area(pixel)	
	YOLOv8	Mask R-CNN	YOLOv8	Mask R-CNN	YOLOv8	Mask R-CNN
MAE	**1.83972**	1.83979	**6.19958**	8.72383	**152.9066**	168.5477

MAE, Mean Absolute Error

**Figure 9:**
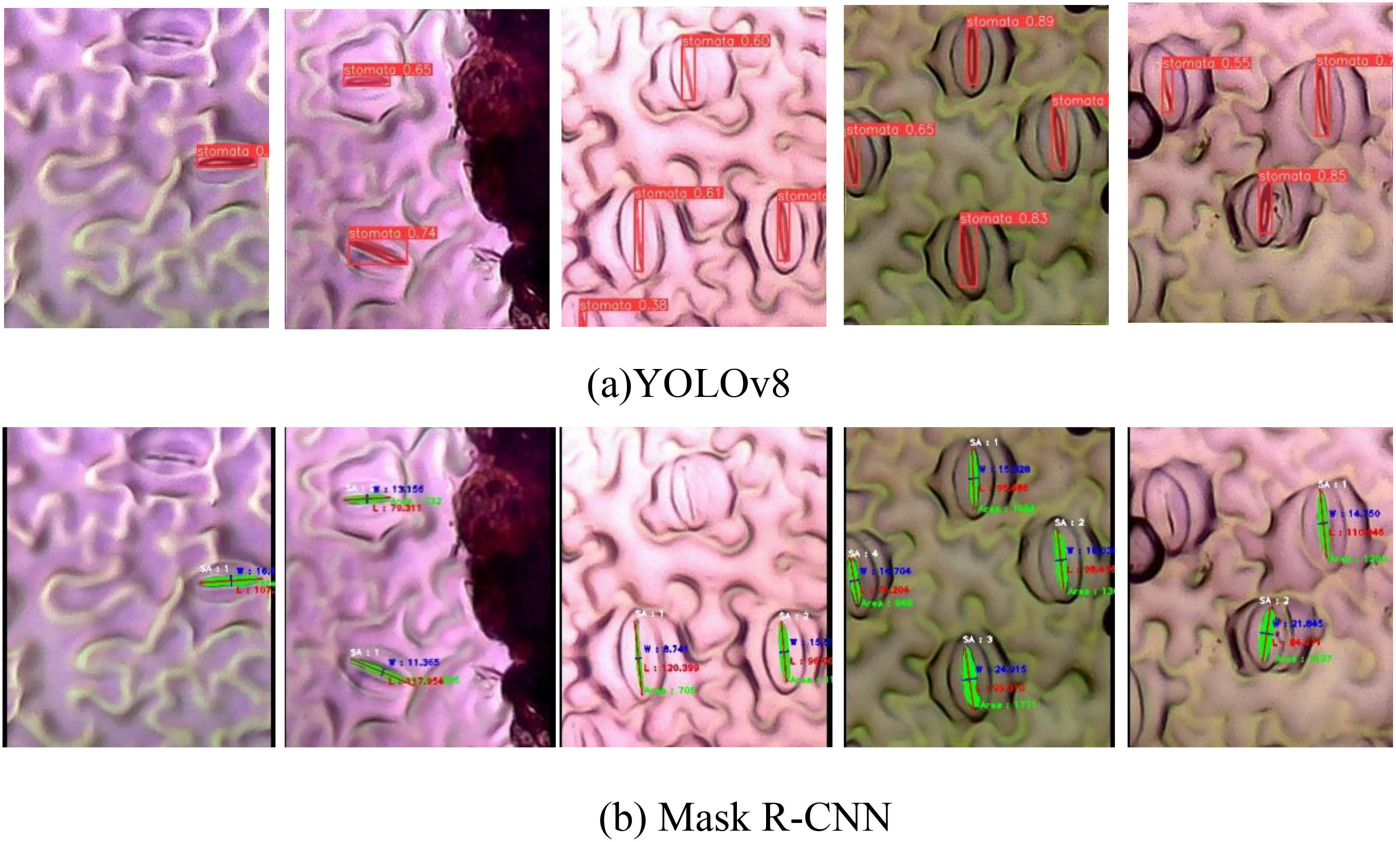
Qualification evaluation for instance segmentation models on the new dataset. **(A)** YOLOv8 predicted stomata pore, **(B)** Mask R-CNN predicted stomata pore.

**Figure 9** illustrates the instance segmentation performance evaluation conducted on the new validation dataset using Mask R-CNN and YOLOv8. In the first row (**Figure 9A**), the predicted stomata pores from YOLOv8 are depicted, while the second row (**Figure 9B**) showcases the predicted stomata pores from Mask R-CNN. The visual analysis reveals that both methods successfully detect clear pores; however, challenges arise in the case of blurry pores. Particularly, YOLOv8 exhibits superior performance compared to Mask R-CNN, as evidenced in the third and fifth columns, where YOLOv8 successfully detects blurry stomata that Mask R-CNN fails to identify. This highlights YOLOv8’s enhanced capability in handling challenging instances, particularly in scenarios involving blurry or less defined stomata structures.

We employed well-established instance segmentation models, Mask R-CNN and YOLOv8, without altering their architectures, the processing times for each model are intrinsically tied to their respective structures. The original developers of YOLO have already provided extensive comparisons of processing times against other methods, including Mask R-CNN. Therefore, we did not perform a separate analysis of processing times in this study.

## 4 Discussion

The comparative analysis of Mask R-CNN and YOLOv8 performance in stomata pore instance segmentation provides valuable insights into the strengths and weaknesses of these state-of-the-art algorithms. Mask R-CNN, a widely recognized instance segmentation method, demonstrated remarkable accuracy in localizing stomatal pores, evident from its mean average precision (mAP) score of 0.97920. This high mAP underscores Mask R-CNN’s ability to precisely outline stomatal regions, making it a robust choice for intricate biological image analysis. In contrast, YOLOv8 exhibited a comparable mAP of 0.98164, slightly surpassing Mask R-CNN. YOLOv8’s precision and recall metrics of 0.94709 and 0.9288, respectively, further emphasize its accurate predictions, ensuring minimal false positives and false negatives. The model’s performance was consistent across varying confidence thresholds, indicating its stability in differentiating stomatal pores from the background.

A notable observation was YOLOv8’s enhanced performance in estimating stomatal pore dimensions. Specifically, YOLOv8 demonstrated lower Mean Absolute Error (MAE) values for width, length, and area predictions compared to Mask R-CNN. This superior accuracy in dimension estimation is crucial for botanical studies, enabling precise quantification of stomatal characteristics.

The blurriness of stomatal images posed a challenge that both models navigated differently. Mask R-CNN showcased robustness, achieving a fine balance between precision (0.95098) and recall (0.95755) even in the presence of blurry or partially visible stomatal pores. YOLOv8, while excelling in dimension estimation, faced challenges in handling blurry images, leading to a slight compromise in recall.

Our research findings hold profound implications for plant biology research, providing valuable insights into the intricate world of plant structures and their responses to environmental cues. Accurate segmentation of stomatal pores, demonstrated by both Mask R-CNN and YOLOv8, opens new avenues for understanding plant physiological processes. Precise localization and quantification of stomatal pores are crucial for unraveling plants’ adaptive strategies to diverse environmental conditions, including drought, humidity, and temperature fluctuations. The advanced instance segmentation techniques employed in this study allow researchers to explore the complexities of stomatal behavior in unprecedented detail. YOLOv8’s superior accuracy in dimension estimation enables nuanced insights into plant adaptation mechanisms, shedding light on evolutionary processes and ecological interactions within various plant species.

Moreover, the robust performance of Mask R-CNN and YOLOv8 in handling challenging image conditions broadens the scope of botanical research. Scientists can now analyze diverse samples, including those with imperfections, without compromising the integrity of their analyses. This inclusivity is crucial for comprehensive studies aiming to capture the true diversity of stomatal structures across plant species and environmental contexts. Practically, these findings have significant implications for agriculture and ecology. Accurate stomatal pore segmentation is essential for assessing plant water-use efficiency, a critical parameter in crop breeding programs. Understanding stomatal behavior under different environmental stressors is invaluable for optimizing agricultural practices and enhancing crop resilience.

## 5 Conclusions

Our study makes a substantial contribution to the field of plant biology by introducing a unique dataset focused on stomata pore instance segmentation. We conducted a meticulous comparison between two cutting-edge instance segmentation methods, Mask R-CNN and YOLOv8, using this innovative dataset. Notably, our research introduced preprocessing techniques that enhanced the accuracy of these models significantly. Through a comprehensive analysis, YOLOv8 demonstrated superior performance, particularly in the precise calculation of stomata pore parameters. The implications of our findings are significant for biology research. The new dataset and the in-depth comparative analysis provide fundamental insights, opening doors for more nuanced explorations into plant physiology. These insights are valuable for studying plant adaptations, ecological dynamics, and evolutionary biology. Additionally, the proposed preprocessing enhancements offer valuable insights for refining image analysis techniques, not only benefiting plant biology but also finding applications in various scientific domains where precise image segmentation is essential. In essence, our research represents a substantial advancement in unraveling the complexities of plant structures, contributing both to theoretical understanding and practical applications in the scientific community.

## Data Availability

The raw data supporting the conclusions of this article will be made available by the authors, without undue reservation.
